# High-Frequency Percussive Ventilation: A Promising Rescue Strategy in Severe Lung Disease of Extremely Low Gestational Age Neonates

**DOI:** 10.3390/children11101239

**Published:** 2024-10-15

**Authors:** Kevin Louie, Kristina Ericksen, Lance A. Parton

**Affiliations:** Division of Newborn Medicine, Maria Fareri Children’s Hospital at Westchester Medical Center, and New York Medical College, Valhalla, NY 10595, USA; kevin.louie@vumc.edu (K.L.); kericks@bhmcny.org (K.E.)

**Keywords:** HFPV, high-frequency percussive ventilation, ELGANs, extremely low gestational age neonates, severe neonatal lung disease

## Abstract

**Objective:** The aim of this study was to evaluate high-frequency percussive ventilation (HFPV) as a rescue strategy for extremely low gestational age neonates (ELGANs) with severe lung disease. **Methods:** This is a retrospective review of 16 ELGANs with severe lung disease who were placed on HFPV following a lack of improvement on other modes of conventional and high-frequency ventilation. **Results:** The gestational age of these 16 infants was 25 (24, 26) weeks and their birth weight was 640 (535, 773) grams [median (IQR)], with the survivors being more immature compared to those who died [24 (23, 26) and 26 (25, 28) weeks, respectively; (*p* = 0.04)]; and with an overall mortality of 31% (N = 5). Of those who died, 60% were SGA (*p* = 0.02). Following placement on HFPV, the survivors had a statistically significant decrease in their respiratory severity scores (RSSs) [11 (9, 14) to 6 (5, 13), *p* = 0.03] compared to those who did not survive [15 (11, 16) to 11 (6.8, 14.5), *p* = 0.32] due to an improvement in their oxygenation [FiO_2_: 0.95 (0.85, 1) to 0.6 (0.4, 0.9), *p* = 0.01; compared to 1 (1, 1) to 1 (0.7, 1); survivors and non-survivors, respectively; *p* = 0.32]. Chest X-rays also showed significantly improved aeration due to decreased areas of atelectasis in those who survived. **Conclusions:** HFPV may be an appropriate rescue mode of high-frequency ventilation in the ELGAN population with severe lung disease, particularly for patients with impaired oxygenation and ventilation difficulties due to shifting atelectasis and mucous plugging.

## 1. Introduction

High-frequency percussive ventilation (HFPV) has been utilized in neonatal, pediatric, and adult populations since its emergence in the late 1980s. It is a versatile form of high-frequency ventilation that uses time-cycled, flow-regulated ventilation with superimposed percussion throughout the respiratory cycle [1,2]. This allows for the integration of convective, diffusive, and percussive mechanisms of gas exchange. The delivery of high-frequency breaths at a sub-dead space volume is accomplished through a Phasitron^®^, which employs a sliding venturi and utilizes the Bernoulli principle to provide incremental lung recruitment and aid in mobilization of secretions. As the lung inflates, a resulting back pressure is generated, which automatically decreases the entrainment of air towards the patient. This auto-downregulation of flow can assist in limiting over-inflation and barotrauma. Autoregulation is also present during lung recovery, as with improving compliance and decreasing downstream resistance, the lung ‘feedback’ to the venturi device automatically decreases the delivered pressures, reducing the risk for barotrauma. In addition to these high-frequency breaths and increased humidified heated air, a countercurrent laminar flow is created, removing debris and aiding in mucociliary clearance of secretions [1,2,3]. This mechanism may explain the observed benefit in airway burn victims, cystic fibrosis, and ARDS [4,5,6,7]. Furthermore, lung computerized tomography attenuation has revealed increased areas of ventilation in those with ARDS after being placed on HFPV [8].

Some of the earlier applications of HFPV included the management of severe lung disease in the neonatal population [3]. Its popularity in this field arose from a desire to combine the benefits of both conventional and high-frequency ventilation while reducing any associated damage, as demonstrated in animal models [9]. Oxygenation and compliance were preserved, while equivalent lung volumes were maintained at lower mean airway pressures (MAPs) [3], resulting in a lung-protective strategy [9].

The Regional NICU (RNICU) at Maria Fareri Children’s Hospital at Westchester Medical Center has been using HFPV since 2001, most commonly as a rescue modality for those infants with acute respiratory failure. As far as we know, there are no studies using HFPV in extremely low gestational age neonates (ELGANs) with severe lung disease, who have failed multiple ventilation strategies including other forms of high-frequency ventilation (HFV). Therefore, we retrospectively evaluated infants placed on HFPV for severe lung disease to better evaluate the clinical characteristics, and HFPV settings of those ELGAN infants who could be rescued as well as those who did not respond to HFPV interventions.

## 2. Materials and Methods

We performed a retrospective review of ELGAN infants from December 2017 to December 2021, who were placed on HFPV (VDR^®^-4) as a rescue strategy for severe lung disease. This investigation was approved by the IRB of New York Medical College and Westchester Medical Center (#15,343) with the need for written consent waived. These infants were managed in the RNICU at Maria Fareri Children’s Hospital of Westchester Medical Center, a level IV program with a catchment area including over 23,000 births annually. In general, ELGAN infants are managed initially with non-invasive ventilation unless they require intubation and conventional ventilation. However, the ELGAN infants in this cohort were all intubated in the delivery room and placed on conventional mechanical ventilation with an endotracheal tube. Further escalation to either a high-frequency oscillatory ventilator (HFOV) or high-frequency jet ventilator (HFJV) is determined by their clinical needs, as well as chest X-rays, with those demonstrating air leak, pulmonary interstitial emphysema, or asymmetrical lung expansion being placed on HFJV, while those demonstrating increased oxygenation and ventilation needs without such radiologic findings being placed on HFOV. In severe cases, with little response to one of the HFV (high-frequency ventilator) modalities, infants may be trialed on the other HFV.

The decision to use HFPV is based on the discretion of the attending physician, but it is primarily used for the rescue of ELGAN infants with severe lung disease, and/or for those infants with recurrent plugging and/or atelectasis, who have failed or are failing to respond to HFOV and/or HFJV. Initial adjustments to HFPV settings are made according to pulse oximetry and transcutaneous CO_2_ monitors (TCOM), along with blood gases, chest radiographs and thoracic responses to HFPV (‘jiggle’). Strategies for optimizing oxygenation and ventilation are personalized to the infant and their underlying conditions. With the liberation of mucous plugging, the oxygen saturations typically drop initially, while the TCOM settings initially increase, signaling the need to suction secretions from the airway. Following removal of airway secretions, these parameters return to baseline. While we do not routinely collect tracheal aspirates from intubated infants, we do so when there is a change in the quantity or quality of these airway secretions. When tracheitis is diagnosis (with neutrophils in the aspirate), a 5-day course of nebulized tobramycin is prescribed.

Demographics of the ELGAN infants were compiled including birth characteristics, the use of alternative ventilator strategies, and ventilator settings prior to placement and after stabilization on HFPV. The respiratory severity score (RSS) was then calculated by multiplying the FiO_2_ and MAP before and after the changes were made. Sepsis denotes a positive culture (blood, urine, or CSF). Screening for patent ductus arteriosus (PDA) is performed on day 3–5, and those with moderate to large PDAs with left-to-right flow are eligible for parenteral treatment with ibuprofen or indomethacin of up to 3 courses with dosing determined by hour of life [10]. Those with persistent PDAs are procedurally closed (Piccolo or ligation). Fluid administration is given to optimize the nutritional status, with most infants in this study having achieved full enteral feedings. We do routinely use post-natal steroids and diuretics for those with refractory BPD. If there are echocardiographic changes in pulmonary hypertension, inhaled NO and/or sildenafil are administered.

SPSS version 16 was used to assess categorical variables using chi squared or Fisher’s exact tests and continuous nonparametric data using the Mann–Whitney test. The RSS was compared before and after the transition to HFPV and depicted as a box and whisker plot. Infants were dichotomized into those who survived and those who did not survive. Between- and within-group RSS values were analyzed.

## 3. Results

The overall Demographic and Respiratory characteristics are shown in Table 1 and Table 2, respectively. A comparison between the Survivors and Non-Survivors is shown in Table 3. The gestational age of those who survived was younger at 24 (23, 26) weeks compared to those who did not survive at 26 (25, 28) weeks (*p* = 0.04). Their birthweights were not statistically different, although there were more SGA infants in the non-survivors (60%) compared to the survivors (0%) (*p* = 0.02). Of the 16 infants, 11 survived and 5 did not. While all those who died had severe bronchopulmonary dysplasia, two had sepsis [one with multiple organisms, including MSSA (MSSA pneumonia was also present) and Serratia; one with Gram-negative sepsis]; one had severe pulmonary hypertension (PH) with right-heart failure and cor pulmonale; one had post-op necrotizing enterocolitis with PH; and one had newly diagnosed hypertrophic cardiomyopathy.

**Figure 1:**
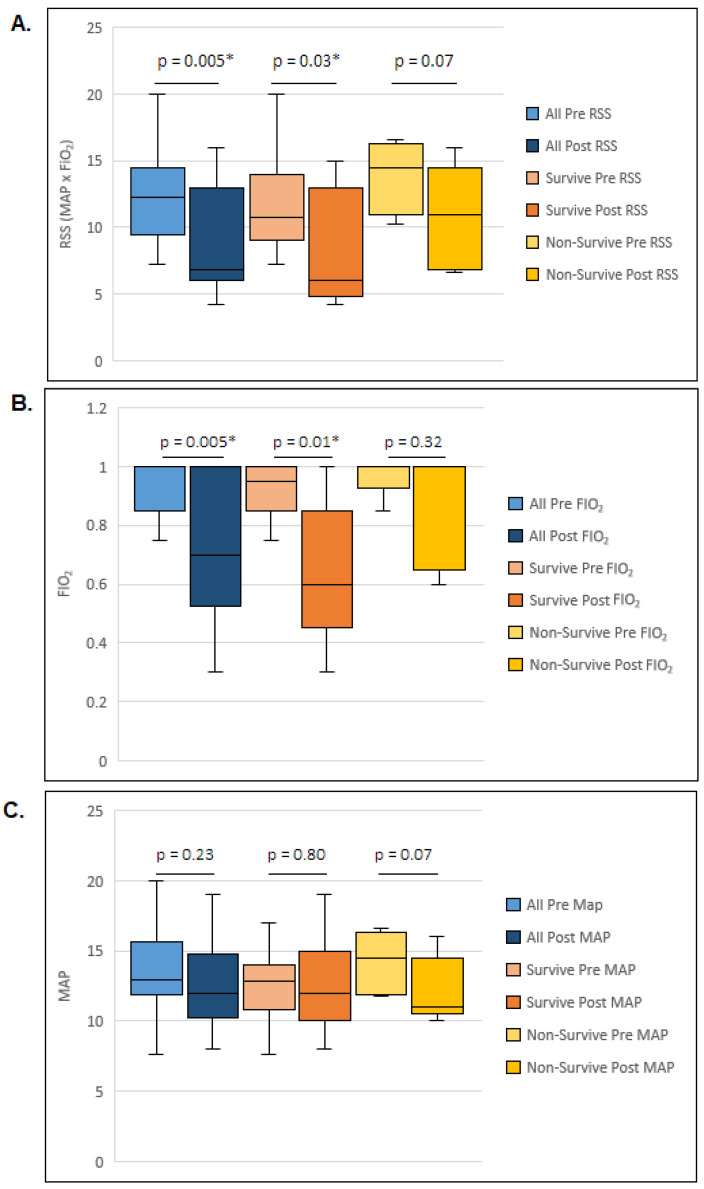
The data summarize the changes for all patients in addition to separating the changes in respiratory severity score (RSS) seen in survivors versus non-survivors. (**A**). Box and whisker plot depicting the RSS before and after being placed on HFPV. (**B**). Box and whisker plot depicting the FiO_2_ before and after being placed on HFPV. (**C**). Box and whisker plot depicting the MAP before and after being placed on HFPV. (* *p* value < 0.05).

All infants were trialed on multiple ventilators with no improvement in their severe lung disease prior to being transitioned to HFPV. Table 4 shows the ventilation and oxygen requirements of the survivors and non-survivors before being placed on HFPV. There were no significant differences between the two groups with respect to the day of life when HFPV was started or stopped, the number of days on HFPV; nor any of the parameters assessed prior to placement on HFPV including: the high frequency ventilator used, the oxygen requirement, the MAP, or the RSS. However, after being placed on HFPV, there was a statistically significant decrease in the RSS from 12 (9, 15) to 7 (6, 13) [median (IQR)] in all patients (Figure 1A; *p* = 0.005), and a significant decrease in the RSS [11 (9, 14) to 6 (5, 13)] [median, (IQR)] in the survivors (Figure 1A; *p* = 0.03). 

Those who survived also had a statistically significant decrease in their FiO_2_ from 0.95 (0.85, 1) to 0.6 (0.4, 0.9) [median, (IQR)] after being placed on HFPV (Figure 1B; *p* = 0.01). There was no statistically significant decrease in their MAPs (Figure 1C). The five infants who died did not show any significant improvement following placement on HFPV. The RSS did not decrease significantly following placement on HFPV, i.e., 15 (11, 16) decreased to 11 (6.8, 14.5) (*p* = 0.32) (Figure 1A), and neither their FiO_2_ nor MAP were statistically different from pre- to post-HFPV.

**Figure 2:**
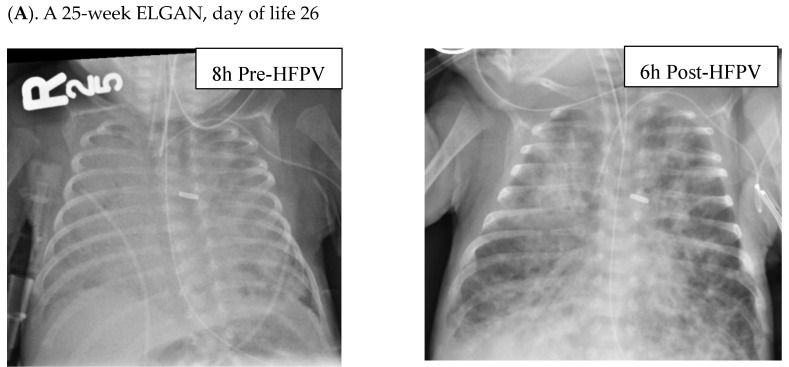

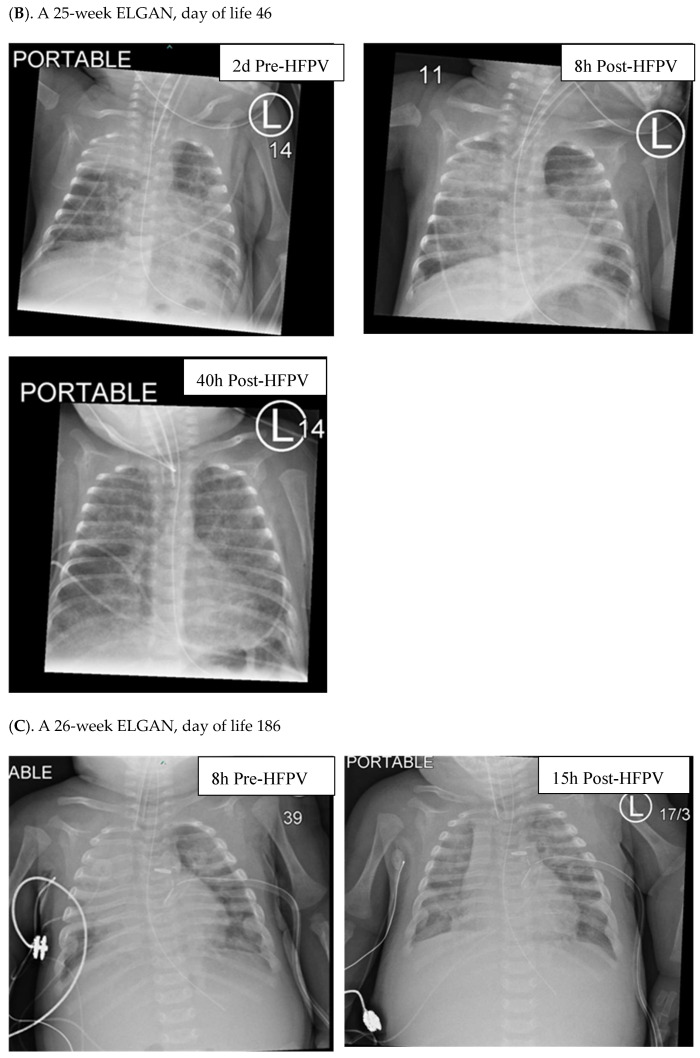
Radiographs of ELGANs placed on HFPV. Patient (**A**) is a 25-week gestation infant who was found to have worsening diffuse atelectasis with almost complete opacification of lung fields on a chest X-ray taken 8 h prior to placement on HFPV on DOL 46. Within 6 h after being placed on HFPV, the infant had a dramatic improvement in his clinical status with decreased FiO_2_ requirements and improved findings on CXR as (shown on the right). Patient (**B**) is a 25-week gestation infant changed to HFPV on DOL 46. CXRs performed 2 days prior, and 8 and 40 h post-placement on HFPV revealed a more gradual clearing of opacified lung fields. Patient (**C**) is a 26-week gestation infant with shifting areas of atelectasis, who was changed to HFPV on d186 (left). CXRs 8 h prior to placement on HFPV and 15 h following HFPV, respectively, revealed shifting and then improved aeration.

Radiographic improvements were seen in ELGANs, with a representative series shown in Figure 2.

## 4. Discussion

There are many challenges associated with achieving adequate oxygenation and ventilation in ELGAN infants with severe lung disease. The respiratory epithelium, vascular endothelium, and extracellular matrices of ELGANs are immature and highly susceptible to environmental exposures such as hyperoxia, inflammation, and mechanical ventilation [11]. Endogenous surfactant is also susceptible to inactivation by these same factors. Following premature birth and environmental exposures, the developmental trajectory of these pulmonary components is also attenuated [12]. We studied a group of ELGANs with respiratory failure due to evolving severe BPD with a multi-compartmentalized lung characterized by impaired oxygenation and ventilation due to gas trapping, atelectasis, and mucous plugging, often complicated by pulmonary hypertension (75%).

The published use of HFPV in the ELGAN population is sparse with limited patient numbers reported [6]. Studies in older gestational age infants have shown decreased oxygen requirements, improved ventilation, and lower mean airway pressures with HFPV [13,14,15]. The use of HFPV is mostly reported in the adult population and to a lesser extent in the pediatric intensive care unit [8,15]. Its use in the NICU is uncommon and to our knowledge, no studies have been published on the ELGAN population who have failed other modes of ventilation, especially HFV. We report our experiences and results, which may be of interest to other providers taking care of this unique population of ELGANs with severe lung disease.

Our approach has been to utilize HFPV in a rescue mode once HFJV and/or HFOV have been optimized, while addressing echocardiographic evidence of pulmonary hypertension with iNO and/or sildenafil and treating sepsis with antibiotics as warranted. Fluid administration to optimize nutrition, and diuretic administration is personalized to the individual patient.

**Figure 3:**
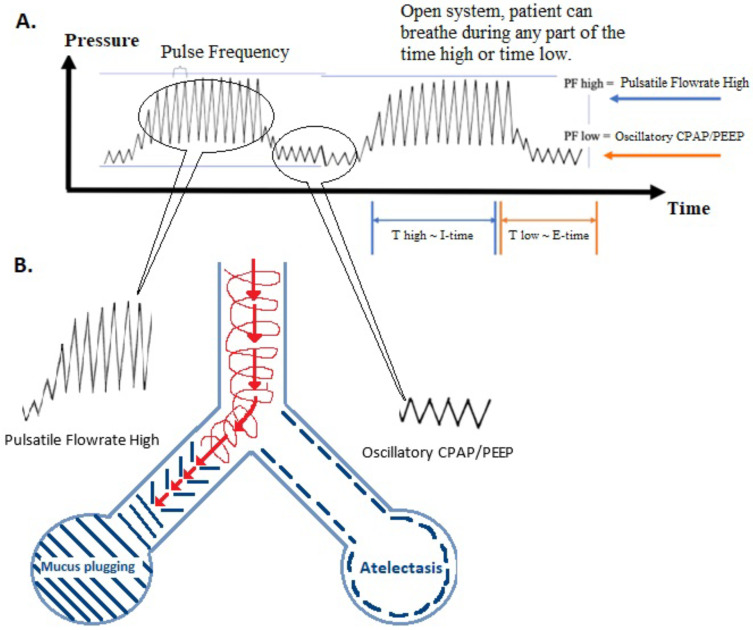
Proposed mechanisms of action of HPFV. The HFPV tracing (**A**) provides percussive, pulsatile flow throughout the ventilated cycle. Convective ventilation is generated between T high and T low (analogous to I- and E-times) and is visible in the patient by chest rise. The maintenance of FRC is supported during percussive pulsatile flow during T low (E-time) at CPAP/PEEP. (**B**) The ability to mobilize secretions is illustrated by the mucous-congested airway, as percussive, penetrating, pulsatile flow is entrained to the center of the airway during PF high and exhaled passively adjacent to the airway walls (corkscrew appearance) during passive exhalation. Atelectasis is illustrated on the other side of this diagram. During oscillatory CPAP/PEEP (PF low), the goal is to minimize atelectasis during continued percussive, pulsatile flow, while maintaining FRC, with minimal impact on venous return.

In HFPV, high-velocity percussive pulses are delivered at two levels of pulsatile flow (PF) and oscillatory CPAP/PEEP [1] (Figure 3). The PF high is achieved following incremental increases in high-velocity flowrates entrained to the center of the patient’s airway, allowing recruitment and penetration of these pulses. The second level of pulsatile flow occurs at the oscillatory CPAP/PEEP, shown as PF low (Figure 3). The level of PF low is adjusted to maintain functional residual capacity (FRC), minimize atelectasis, and improve lung compliance while minimizing hemodynamic compromise, such as impaired venous return. The convective rate is determined by the duration of the PF high, or the T high (time at the high PF); and the duration of PF low, or T low-analogous to I- and E-times. These settings enable passive recoil of the lungs. The pulse frequency is delivered throughout the ventilatory cycle, with enhancement of minute ventilation, including penetration and recruitment at PF high; and preservation of FRC by sustaining CPAP/PEEP at PF low, while minimizing the impact on venous return.

HFPV is an ‘open’ ventilator that optimizes gas exchange in a multi-compartmental lung model that contains regions of variable resistance and compliance. The ‘healthier’ regions of the lung are more compliant and have reduced resistance, permitting faster ventilation at lower pressure, while the opposite is true for more ‘diseased’ regions. Specifically, areas of mucous plugging can be targeted by HFPV as the pulsatile flow rate is focused on penetrating these plugs, thereby offering a pathway for removal. On the other hand, atelectasis can be overcome during HFPV by focusing our attention on PF low (Figure 3).

For the infants who survived, there was a dramatic improvement in the RSS due to an improvement in oxygenation following placement on HFPV. Chest X-ray findings for these infants showed significant areas of increased aeration. This indicates an improved functional residual capacity in relation to the effectiveness of HFPV in alveolar recruitment. The infants who died did not show a significant reduction in the RSS. This suggests that other non-respiratory mechanisms may have been responsible for the poor responses to ventilation strategies, such as vascular, cardiac, or inflammation/sepsis. Indeed, two of the non-survivors were infected at the time of death, two others had severe PH, and one had newly diagnosed hypertrophic cardiomyopathy. Interestingly, those who died were more mature (older gestational ages) compared to the survivors, while 60% of the non-survivors were SGA, suggesting that a further growth restriction of the respiratory epithelium, vascular endothelium and/or extracellular matrix may have contributed to pulmonary hypoplasia in these non-surviving ELGAN infants, making rescue more problematic.

We acknowledge the limitations of this retrospective, non-controlled case series. This study was conducted at a single-level IV regional NICU, which serves as a high-risk delivery center for the region. Approximately 50% of the offspring of mothers who deliver at this center will be admitted to the NICU. Therefore, our results may not be applicable to the general ELGAN population. Additionally, these are small patient numbers, which may have affected the overall outcome. While some may argue that the prior ventilator strategies were not optimized prior to placement on HFPV, most of these ELGANs were on 100% oxygen, high MAPs, high-frequency ventilation (HFOV and/or HFJV), and had been trialed on iNO, with limited improvement. In addition, the MAPs from the various HFVs (and therefore the RSS) may not be comparable, as measurements of MAP may be performed at different sites along the ventilator/airway interface. In addition to the limitations in assessing MAP, the lack of arterial sampling in these patients limits the direct comparison of PaO_2_, as well as our capability of calculating oxygenation indices. We acknowledge that scoring the severity of CXRs may have been a helpful comparator. We found a high degree of variability in TCOM data, both within and between patients, and did not collect nor compare these data, although this may have useful in understanding underlying pathophysiological mechanisms of gas exchange. However, we feel this study adds to the sparse literature of HFPV as studies in ELGANs are few with small numbers and none compare HFPV to other modes of high-frequency ventilation, particularly in ELGANs with severe lung disease.

## 5. Conclusions

While the full breadth of uses for HFPV in the neonatal population may not be fully appreciated, we propose that it may be a beneficial rescue strategy in ELGAN infants with severe lung disease characterized by impaired oxygenation and ventilation caused by shifting atelectasis due to extensive mucus plugging. SGA ELGAN infants are the least responsive to rescue from severe lung disease with HFPV. We also propose using HFPV earlier in management instead of primarily as rescue HFV. A randomized controlled trial evaluating the long-term benefits of HFPV compared to other forms of HFV is needed.

## Figures and Tables

**Table 1:** Demographics for all infants.

Total	
N = 16	
Birth Weight (median, IQR)	640 (535, 773)
Gestational Age (median, IQR)	25 (24, 26)
C/S (n, %)	16 (100%)
Male (n, %)	8 (50%)
SGA (n, %)	3 (19%)
Prenatal Steroids (n, %)	15 (94%)
Surfactant (n, %)	16 (100%)
APGAR 1 min ≤ 7 (n, %)	15 (94%)
APGAR 5 min ≤ 7 (n, %)	10 (63%)
Sepsis (n, %)	9 (56%)
PDA (n, %)	15 (94%)
IVH Grade 3/4 (n, %)	1 (6%)
Pneumothorax (n, %)	2 (13%)
iNO (n, %)	12 (75%)
HFJV (n, %)	15 (94%)
HFOV (n, %)	12 (75%)

C/S, caesarean section; SGA, small for gestational age; PDA, patent ductus arteriosus; IVH, intraventricular hemorrhage; iNO, inhaled nitric oxide; HFJV, high-frequency jet ventilation; HFOV, high-frequency oscillatory ventilation.

**Table 2:** Respiratory characteristics for all infants.

Total	
N = 16	
DOL when HFPV was Started (median, IQR)	46 (27, 61)
DOL when HFPV was Stopped (median, IQR)	86 (52, 110)
Days on HFPV (median, IQR)	40 (12, 64)
Pre-HFPV FiO_2_ (median, IQR)	1 (0.85, 1)
Post-HFPV FiO_2_ (median, IQR)	0.7 (0.53, 1)
Pre-HFPV MAP (median, IQR)	13 (12, 16)
Post- HFPV MAP (median, IQR)	12 (10, 15)
Pre-HFPV RSS (median, IQR)	12 (9, 15)
Post-HFPV RSS (median, IQR)	7 (6, 13)
RSS Change (median, IQR)	3 (0.65, 8)

DOL, day of life; HFPV, high-frequency percussive ventilation; MAP, mean airway pressure; RSS, respiratory severity score.

**Table 3:** Survived and non-survived demographics.

	Survived	Non-Survived	*p* Value
	N (11)	N (5)	
Birth Weight (median, IQR)	650 (610, 790)	520 (345, 772)	0.36
Gestational Age (median, IQR)	24 (23, 26)	26 (25, 28)	0.04 *
C/S (n, %)	11 (100%)	5 (100%)	1
Male (n, %)	6 (55%)	2 (40%)	1
SGA (n, %)	0 (0%)	3 (60%)	0.02 *
Prenatal Steroids (n, %)	11 (100%)	4 (80%)	0.31
Surfactant (n, %)	11 (100%)	5 (100%)	1
APGAR 1 min ≤ 7 (n, %)	10 (91%)	5 (100%)	1
APGAR 5 min ≤ 7 (n, %)	8 (73%)	2 (40%)	1
Sepsis (n, %)	5 (45%)	4 (80%)	0.31
PDA (n, %)	10 (91%)	5 (100%)	1
IVH Grade 3/4 (n, %)	1 (9%)	0 (0%)	1
Pneumothorax (n, %)	1 (9%)	1 (20%)	1
iNO (n, %)	8 (73%)	4 (80%)	0.51
HFJV (n, %)	10 (91%)	5 (100%)	1
HFOV (n, %)	7 (64%)	5 (100%)	0.25

C/S, caesarean section; SGA, small for gestational age; PDA, patent ductus arteriosus; IVH, intraventricular hemorrhage; iNO, inhaled nitric oxide; HFJV, high-frequency jet ventilation; HFOV, high-frequency oscillatory ventilation. (* *p* < 0.05).

**Table 4:** Survived and non-survived respiratory characteristics.

	Survived	Non-Survived	*p* Value
	N (11)	N (5)	
DOL when HFPV was Started (median, IQR)	46 (30, 61)	41 (19, 123)	0.72
DOL when HFPV was Stopped (median, IQR)	69 (50, 105)	90 (50, 205)	0.49
Number of Days on HFPV (median, IQR)	38 (11, 45)	66 (21, 84)	0.19
Pre-HFPV FiO_2_ (median, IQR)	0.95 (0.85, 1)	1 (1, 1)	0.31
Post-HFPV FiO_2_ (median, IQR)	0.6 (0.4, 0.9)	1 (0.7, 1)	0.12
Pre-HFPV MAP (median, IQR)	13 (10.8, 14)	15 (11.9, 16.3)	0.49
Post-HFPV MAP (median, IQR)	12 (10, 15)	11 (10.5, 14.5)	0.89
Pre-HFPV RSS (median, IQR)	11 (9, 14)	15 (11, 16)	0.17
Post-HFPV RSS (median, IQR)	6 (4.8, 13)	11 (6.8, 14.5)	0.12
RSS Change (median, IQR)	3 (0, 8.5)	2 (0.7, 6.3)	0.77
HFJV (n, %)	10 (91%)	5 (100%)	1
HFOV (n, %)	7 (64%)	5 (100%)	0.25

DOL, day of life; HFPV, high-frequency percussive ventilation; MAP, mean airway pressure; RSS, respiratory severity score; HFJV, high-frequency jet ventilation; HFOV, high-frequency oscillatory ventilation.

## Data Availability

The original contributions presented in the study are included in the article, further inquiries can be directed to the corresponding author.
